# Hybridization Chain Reactions Targeting the Severe Acute Respiratory Syndrome Coronavirus 2 (SARS-CoV-2)

**DOI:** 10.3390/ijms21093216

**Published:** 2020-05-01

**Authors:** Tzu-Heng Wu, Chia-Chen Chang, Ching-Hsu Yang, Wei-Yin Lin, Tan Joy Ee, Chii-Wann Lin

**Affiliations:** 1 Department Biomedical Engineering, National Taiwan University, Taipei 10617, Taiwan; aresation@gmail.com (T.-H.W.); emily110144@gmail.com (W.-Y.L.); vickyjoytan@gmail.com (T.J.E.); 2 Department of Medical Biotechnology and Laboratory Sciences, College of Medicine, Chang Gung University, Taoyuan 33302, Taiwan; chang@mail.cgu.edu.tw; 3 Graduate Institute of Bio-Electronics and Bio-Informatics, National Taiwan University, Taipei 10617, Taiwan; d02945009@gmail.com; 4 Biomedical Technology and Device Research Laboratories, Industrial Technology Research Institute, Hsinchu 30011, Taiwan

**Keywords:** SARS–CoV-2, hybridization chain reaction, algorithm

## Abstract

In this work, hybridization chain reactions (HCRs) toward *Severe Acute Respiratory Syndrome Coronavirus 2* (*SARS–CoV-2*) nucleocapsid phosphoproteins gene loci and human RNase P are proposed to provide an isothermal amplification screening tool. The proposed chain reactions target the complementary DNA (cDNA) of *SARS–CoV-2*, with loci corresponding to gold-standard polymerase chain reaction (PCR) loci. Four hybridization chain reaction reactions are demonstrated herein, targeting *N1/N2/N3* loci and human *RNase P*. The design of the hybridization chain reaction, herein, is assisted with an algorithm. The algorithm helps to search target sequences with low local secondary structure and high hybridization efficiency. The loop domain of the fuel hairpin molecule H1 and H2, which are the tunable segments in such reactions, are used as an optimization parameter to improve the hybridization efficiency of the chain reaction. The algorithm-derived HCR reactions were validated with gel electrophoresis. All proposed reactions exhibit a hybridization complex with a molecular mass >1.5k base pairs, which is clear evidence of chain reaction. The hybridization efficiency trend revealed by gel electrophoresis corresponds nicely to the simulated data from the algorithm. The HCR reactions and the corresponding algorithm serve as a basis to further *SARS–CoV-2* sensing applications and facilitate better screening strategies for the prevention of on-going pandemics.

## 1. Introduction

Starting from December 2019, the new *Severe Acute Respiratory Syndrome Coronavirus 2* (noted as *SARS–CoV-2* hereafter) has led to a major pandemic around the globe. Although the reverse transcriptase–polymerase chain reaction (RT–PCR) serves as a gold standard for nucleic acid screening of SARS–CoV-2, novel screening methodologies with less requirements of materials and equipment are still needed, considering a large number of patients for screening in the current stage of the outbreak. In this perspective, PCR serves better as a diagnostic tool than a screening tool, due to the power-consuming thermal cycler and its need for Taq polymerase. On the contrary, hybridization chain reaction does not require enzymes for polymerization processes and can be carried out at room temperature. Therefore, such an isothermal detection method could be a good candidate for a screening tool for such scenarios.

HCR was firstly proposed by Dirk et al. in 2004 [1], and later on, applied to various detection targets [2,3]. The HCR is, in essence, a type of room temperature isothermal DNA chain amplification method. There are three major molecular components in an HCR reaction: a target gene segment and two fuel hairpins (noted as H1 and H2). The characteristics of H1 and H2 are their short toehold segment at the 5′ and 3′ ends, respectively. In the first stage of the reaction, driven by toehold-mediated strand displacement reaction [4], H1 hybridizes with the target gene segment. The H1-target double-strand complex leaves a short segment of sequence that is complementary to the toehold of the H2. Therefore, H2 hybridizes with the complex again. Due to the delicate design of HCR, the hybridized H2 opens its hairpin and leaves a new segment which has a sequence identical to the target. From this point on, a chain reaction of hybridizations is initiated and will continue to grow as long as the fuel hairpins remain. The advantage of the HCR is that it does not require a thermal cycler or enzymes for amplification. Moreover, HCR can be performed at room temperature without problems. These attributes make HCR a good candidate for rapid screening, with a detection limit down to femtomolar [5,6].

We propose herein a set of HCR reactions toward *SARS–CoV-2*. The HCR reactions are designed toward *N1/N2/N3* cDNA of *SARS–CoV-2* and Human *RNase P* gene loci. These gene loci correspond to the RT–PCR probe zone for *SARS–CoV-2* detection, as issued by the Center for Disease Control (CDC) of the United States (https://www.cdc.gov/coronavirus/2019-ncov/lab/rt-pcr-detection-instructions.html). The *N1/N2/N3* loci correspond to different segments on *nucleocapsid phosphoproteins* gene (commonly noted as *N* gene) of the *SARS–CoV-2*, while human *RNase P* is commonly used as a control set. According to the US CDC, the *N1* and *N2* probes are highly specific to *SARS–CoV-2*, while the *N3* target segment is more universal for the detection of a corona-like virus. We propose herein HCR reactions targeting the virus cDNA so that they can be incorporated into commercially available viral cDNA synthesis kits. In this way, this HCR-based *SARS–CoV-2* screening tool may better fit the current screening route. The second reason for choosing cDNA as the target rather than RNA is that DNA has better stability and shelf life.

A simple algorithm, named “HCR designer”, is devised to assist in-silico design of HCR. HCR designer helps to analyze target sequence, to generate the corresponding H1/H2, and to estimate the HCR efficiency. The core of the algorithm is a combination of python local calculation script with web a server tool, via the Selenium package. Through this HCR designer, it is possible to access different design parameters in a simple and automated fashion, facilitating faster HCR design and study.

## 2. Methodology

The hybridization chain reactions were validated using gel electrophoresis herein. The HCR firstly took place in reaction tubes under 1× PBS with 0.5 M NaCl (Sigma-Aldrich, USA) under room temperature. Such salt concentration is found to optimize the rate of reaction. Further increase of salt concentration up to 1× PBS with 1 M NaCl is found to significantly reduce the reaction rate despite the seemingly beneficial charge screening effect of higher ionic strength. This is likely due to the presence of a self-stable target sequence under higher salt concentrations. The H1 and H2 were firstly mixed in equal concentration, then the target strand was added into the solution for 30 min reaction time. The final concentration of H1 and H2 was 5 μM, and the concentration of the target was varied to demonstrate the HCR, as suggested by the first work reported by Dirk et al. [1]. The running gel for electrophoresis was made of 2% agarose in 1× sodium borate buffer (1× SB buffer) (Sigma-Aldrich, USA). The electrophoresis was done with a 100-V driving voltage (with MAJOR SCIENCE MP-100) at room temperature, using a 1× SB running buffer. The fluorescent dye used for imaging was Safe Green (Hycell, Taiwan). The concentrations of oligonucleotides used in gel electrophoresis were set to provide better fluorescence imaging results.

## 3. Results

**Figure 1:**
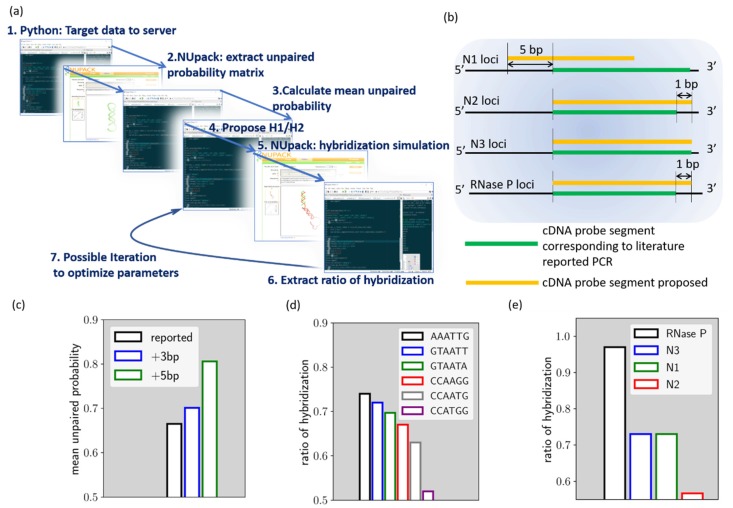
(**a**) Simplified scheme of the proposed HCR designer. (**b**) Comparison between the proposed HCR probe loci and cDNA loci corresponding to the US Center for Disease Control PCR probe. (**c**) mean unpaired probability for target sequence from different loci of the *N1* gene. (**d**) The calculated *r_h_* for *N1* HCR with different H1/H2 loop domains. (**e**) The calculated *r_h_* value for all proposed HCR reactions.

The HCR evaluation algorithm was built using open-source Python 3.0 with Spyder IDE environment from Anaconda (Anaconda 3, Anaconda Inc., Texas, USA),. A brief scheme of the algorithm is shown in Figure 1a. Through the Selenium package, the python script connects to the NUpack web server (http://www.nupack.org/partition/new) to inquire about structural or hybridization simulation data. The use of the Selenium package allows our local calculation script to leverage the web server service simulation data, which can help to quickly assemble new numerical tools from different services. In the HCR designer algorithm, the target gene segment is firstly sent to NUpack to extract the unpaired probability of the bases [7]. With the extracted probability array, the HCR designer can then calculate the mean unpaired probability of the target sequence. The mean unpaired probability is used herein as a numerical indicator of the presence of local secondary structure. In HCR reaction, such a secondary structure on the target should be avoided since it tends to reduce overall kinetics of HCR as the target becomes more and more self-stable. Once an optimized target sequence is determined, the script proposes H1/H2 hairpins for the corresponding HCR reaction. In the proposition of HCR reactions, different loop domains can be proposed to yield several sets of HCR. HCR designer then sent the target/H1/H2 to NUpack again to estimate the chain reaction efficiency. To evaluate the chain hybridization efficiency, H1/H2 and target are pooled together and allowed to hybridize with each other in the simulation. The maximum coupling strands for the simulation is set at 3, since the intention of the script is to indicate the interaction efficiency between the three entities, instead of providing an exact solution. The hybridization of the target, H1, and H2, is the fundamental reaction that is repeated throughout the HCR process and should, therefore, be able to be used as a numerical indicator of the chain reaction efficiency. The simulation then gives the final output of the algorithm, the ratio of hybridization (r_h_), which is defined as

(1) $$r_{h}=\frac{C_{t12}}{C_{t}}$$

where C_t12_ is the concentration of the target–H1–H2 complex, C_t_ is the initial concentration of the target at the beginning of the reaction. $r_{h}$ is then used as the indicator for hybridization efficiency. Limited by the scope and length of the article, the algorithm flowchart and the details of the data processing are put in supplementary information for interested readers (Cf. Figure S1 and Figure S2). Readers may also download the full HCR designer script from our dedicated website (http://hcrd.plasmonictron.com/index).

With the assistance of the designer program, HCR for *N1/N2/N3* and *RNase P* is proposed and shown in Table 1.

Compared to the probe segment currently used for PCR loci, some modifications were introduced in the HCR targets. As will be described later, we have modified the loci of *N1* to reduce the local secondary structure. Modifications were also made to all the targets, so that they are of equal 24 base length, in contrast to PCR probes. With identical target sequence lengths but a difference in base composition, the electrophoresis results of these HCR reactions can serve as preliminary validation data for the algorithm.

These minor modifications are noted in Figure 1b. As shown by the figure, the N1 cDNA loci are shifted by 5 base pairs toward the 5′ end of the cDNA, compared to the cDNA loci corresponding to the PCR. As for the *N2*, *N3*, and *RNase P* cDNA loci, they are not different from the PCR loci, while *N2* and *RNase P* target sequences are extended by 1 base pair. In this way, all target sequences are of 24 base pairs in length.

Figure 1c–e reveals the process of our algorithm, using the proposed *N1* HCR as an example. As discussed earlier, the algorithm, parsing the target sequence simulation data from NUpack, indicates the mean unpaired probability. We compare herein the mean unpaired probability of the *N1* cDNA target that corresponding to the PCR probe (noted as reported loci), the loci further shifted by 3 base pairs toward cDNA 5′ end (noted as +3 bp loci) and the loci shifted by 5 base pairs toward cDNA 5′ end (noted as +5 bp loci). As shown in Figure 1c, the result indicated that the +5 bp loci have a mean unpaired probability of around 81%, which is the best option among the three loci. This locus was therefore chosen as the *N1* HCR target. In the next step, we try to proposed different loop domains for fuel hairpins and evaluate hybridization efficiency. As can be seen from Figure 1d, we evaluated r_h_ for six different ad-hoc loop sequences. As indicated, the r_h_ value is strongly affected by the loop domain choice.

The r_h_ of *N1* HCR for ’AAATTG’ loop domain is 0.73, while it can be as low as 0.52 if ’CCATGG’ loop domain is chosen. Therefore, ’AAATTG’ loop domain is selected for *N1* HCR. The above procedures were also carried out for the proposed *N2*, *N3*, and *RNase P* target genes and thus generated the proposed HCR reactions (Cf. Table S1).

**Figure 2:**
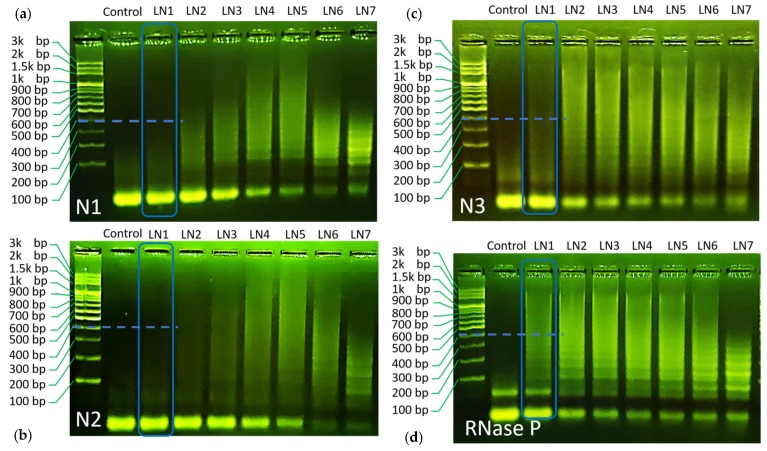
(**a**) Gel electrophoresis result for the proposed *N1* HCR. (**b**) Gel electrophoresis result for the proposed *N2* HCR. (**c**) Gel electrophoresis result for the proposed *N3* HCR. (**d**) Electrophoresis result for the proposed *RNase P* HCR.

Gel electrophoresis was used herein to validate the proposed HCR reaction. Gel electrophoresis results are all repeated for at least three times to confirm the observed trend, and typical results are shown in Figure 2a–d. The electrophoresis experiments were made with 8 lanes (noted as LN in the image), composed of a control group (no target), LN1 (H1:H2:target = 10:10:1), LN2 (H1:H2:target = 5:5:1), LN3 (H1:1H2:target = 3.3:3.3:1), LN4 (H1:1H2:target = 2.5:2.5:1), LN5 (H1:1H2:target = 2:2:1), LN6 (H1:1H2:target = 1:1:1), and LN7 (H1:1H2:target = 1:1:2).

As can be seen through the LN1–LN7 of each panel, all designed reactions have shown chain hybridization with a maximum molecular weight over 1.5 k base pair under a proper condition. These results indicate the success of the designed HCR reaction towards *SARS–CoV-2* gene detection.

Using gel electrophoresis data, the values of r_h_ were evaluated for its role as an indicator of HCR efficiency. For this purpose, we compared LN1 for all HCR reactions. In this way, all reactions are set under the same H1/H2/target concentrations and can be compared on fair grounds. It can be seen from the gel image that the trend of hybridization efficiency follows the order of *RNase P* > *N3~N1* > *N2*, respectively (*Cf.* the blue dashed marker line for each HCR reaction, a more detailed comparison can be found in supplementary information) (Cf. Figure S3). Correspondingly, the simulated r_h_ values follow similar trends, as shown in Figure 1e. Only a minor discrepancy was found for *N3* and *N1* HCR panel. While the calculated r_h_ value was similar for both reactions, *N3* demonstrated slightly higher efficiency as compared to *N1* in gel electrophoresis.

## 4. Conclusions

HCR reactions toward cDNA of *SARS–CoV-2*, targeting *N1/N2/N3* loci on *SARS–CoV-2 N* genes and human *RNase P*, have been proposed and demonstrated. The loci chosen for the HCR were near the corresponding PCR loci proposed by US CDC, while slight modifications were made to provide better HCR efficiency. A simple algorithm, the “HCR designer”, was proposed for the in-silico design of HCR, combining Python local script and the capacity of the Selenium package to leverage data from web server NUpack. Assisted by this algorithm, the local secondary structure of the target sequence was analyzed and minimized through the use of the mean unpaired probability as a numerical indicator. Then, different hairpin H1/H2 loop domains were tested in-silico in search of better hybridization efficiency. *N1* gene-targeted HCR was used herein to demonstrate the algorithm, where six different loop domains were tested in the simulation, with the best hybridization ratio reaching a value of 0.81. The algorithm-derived HCR reactions were then validated by gel electrophoresis. The results indicated that all proposed reactions can provide amplification products with molecular weight >1.5 k base pair under proper conditions. We compared, in a semi-quantitative fashion, the hybridization efficiency inspected from the gel electrophoresis image and the calculated r_h_ value. The gel electrophoresis results reveal a hybridization efficiency trend with *RNase P* > *N3*~*N1* > *N2*, which is highly similar to the calculated r_h_ value. These results preliminarily reveal the efficacy of the proposed HCR designer. The proposed *SARS–CoV-2* HCR can be widely applied in conjunction with different sensing strategies, such as fluorescence, gold nano-particle based colorimetry [8,9], or surface plasmon resonance biosensor [10]. The proposed algorithm provides an easy design of HCR targets and conditions, which can facilitate HCR applications.

Finally, we would like to point out the potential of such an algorithm structure, combining local script with the Selenium package to leverage different web server services. First, despite the fact that the target sequence in the algorithm was here limited to a short segment, for the purpose of comparison with PCR probes, the HCR designer can easily carry out tedious analysis of a full target gene sequence and then allocate a domain exhibiting the best performance. Secondly, with automated interaction with a web server, further features can be incorporated to provide a deeper insight into the HCR design. For example, the optimized target sequence can be further sent to the BLAST server to analyze the specificity of the target among the different lineages of the virus gene. Considering the lack of a hybridization chain reaction design web server, our open source HCR designer algorithm should be beneficial to HCR studies in the near future. Finally, it would be interesting to further enhance such an algorithm with kinetic data measured by quantitative biosensors such as surface plasmon resonance to provide a quantitative and sophisticated HCR prediction model.

## Figures and Tables

**Table 1:** Fuel hairpin molecule H1/H2 and target sequence chosen for the SARS–CoV-2 hybridization chain reaction.

cDNA Target	Target	H1	H2
*N1*	5′-CTGAG GGTCC ACCAA ACGTA ATGC-3′	5′-GCATTACGTT TGGTGGACCC TCAGAAATTG CTGAGGGTCC ACCAAACG-3′	5′-CTGAGGGTCC ACCAAACGTA ATGCCGTTTG GTGGACCCTC AGCAA TTT -3′
*N2*	5′-CTGAA GCGCT GGGGG CAAAT TGTG-3′	5′-CACAATTTGC CCCCAGCGCT TCAGACTATG CTGAAGCGCT GGGGGCAA-3′	5′-CTGAAGCGCT GGGGGCAAAT TGTGTTGCCC CCAGCGCTTC AGCATAGT-3′
*N3*	5′-CAGGA TTGCG GGTGC CAATG TGAT-3′	5′-ATCACATTGG CACCCGCAAT CCTGCCTGGT CAGGATTGCG GGTGCCAA-3′	5′-CAGGATTGCG GGTGCCAATG TGATTTGGCA CCCGCAATCC TGACCAGG-3′
*RNase P*	5′- GTTCT GACCT GAAGG CTCTG CGCG -3′	5′-GTTCTGACCT GAAGGCTCTG CGCGTCTAGT CGCGCAGAGC CTTCAGGT-3′	5′-CGCGCAGAGC CTTCAGGTCA GAACACCTGA AGGCTCTGCG CGACTAGA-3′

## Supplementary Materials

Supplementary materials can be found at https://www.mdpi.com/1422-0067/21/9/3216/s1.

## Abbreviations

HCR	Hybridization Chain Reaction
