# Supplementary material for: Hybridization Chain Reactions Targeting the Severe Acute Respiratory Syndrome Coronavirus 2 (SARS-CoV-2)
Source: Int J Mol Sci. 2020 May 1;21(9):3216. doi: 10.3390/ijms21093216 (PMC7246904; doi:10.3390/ijms21093216)
Supplement: Supplementary file 1 [file ijms-21-03216-s001.pdf]

International Journal of
Molecular Sciences

Supplementary

S1. HCR Algorithm Flowchart

In this section, the algorithm of the HCR designer script is thoroughly introduced. We have also allocated a website to download the HCR designer, where installation guide and solutions to technical issues can be found (http://hcrd.plasmonictr on.com/index).

The flow chart of the HCR designer can be seen in Figure S1. HCR designer takes three types of input, including:

-a raw sequence data

-loop domain sequence of user's choice

-simulation conditions

The "raw sequence data" herein refers to the sequence that the user enters into the script for analysis. The raw sequence, i.e., the target of the HCR, is 24 base pairs long. The user also needs to enter the HCR simulation conditions, which are the NaCl concentration and temperature of the experiment. Once the data has been entered, the HCR designer will first send the raw gene sequence to the NUpack server, using Selenium, to perform secondary structure analysis. Once the simulation is complete, the script then extracts the structural information data. The structural information data herein refers to the unpaired probability for each base pair, in an array format. Using the unpaired probability array, the script can derive the mean unpaired probability (MUP) for the target sequence. The MUP then give user a simple assessment on the level of intra-molecular hybridization in the target sequence. Based on the result, the user can then decide if such raw sequence is a good candidate for HCR.

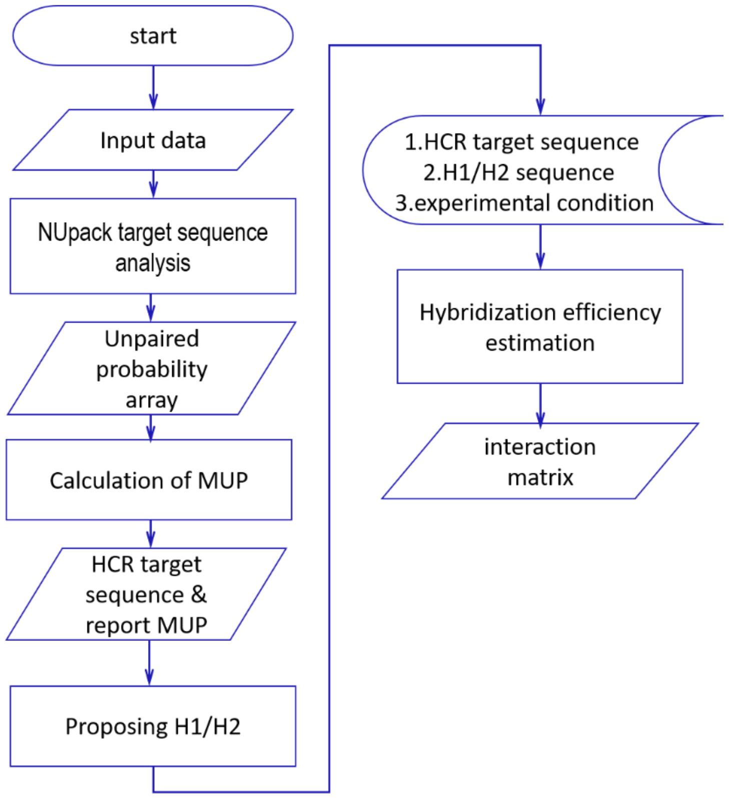

Figure S1. The flowchart of the HCR designer python script.

With the chosen HCR target, the HCR designer then proposes the corresponding H1/H2 sequence, based on the loop domain given by the user. The proposition of the H1 and H2 is a fairly simple process when target sequence and loop domain are pre-determined. This is due to the fact that H1 and H2 are only composed of segments from target sequence and loop domain as shown in Figure S2. As shown in the figure, assuming that the target is composed of *a′* segment followed by ***c′*** segment, and loop domain has a sequence of ***b***, it is clear that H1 and H2 sequence are only composed of segment from a′, b, c′ or their complementary strands. Therefore, H1/H2 can be readily decided in the HCR designer.

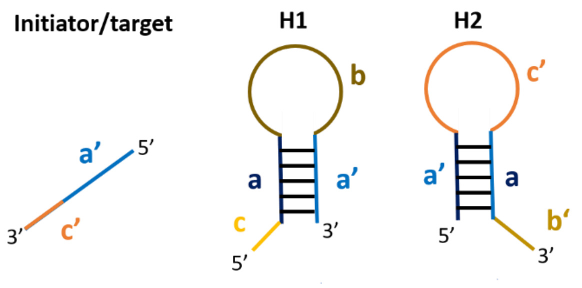

Figure S2. The sequence relationship between target, H1 and H2.

After the HCR target and fuel hairpins are all secured, the HCR designer then sends all sequence information back to the NUpack server. At this stage, the main purpose of the simulation is to estimate the ratio of hybridization of the proposed HCR. In this simulation, the targets, H1 and H2 are allowed to interact with each other. The experimental conditions are set based on the user input, while the maximum interaction strands set to three. The default interaction concentration for strands is set at 1 µM. As mentioned in the article, the purpose of the hybridization simulation here is to indicate the fundamental interaction between three key molecules in this reaction, rather than to provide an exact solution for the HCR efficiency. This gives a simple and intuitive indicator for the user to evaluate the performance of the proposed HCR. Once the simulation is done, the HCR designer extract the "interaction matrix" of the simulation. The "interaction matrix" contains the concentration information for all the existing strands at equilibrium. An example of the interaction matrix for proposed *N1* targeting HCR is shown in Table S1. In each row, following the convention of the NUpack, the first three columns of the table indicate the presence of the given strand. "1" indicates the presence of the given strand in the interaction under study, while "0" indicates the absence of the strand.

For example, the first row in the Table S1 indicates the successful hybridization of target/H1/H2 with a concentration of 0.7341 µM. Considering the 1 µM starting concentration of the target, the ration of hybridization (r_h) is therefore 73.41% as defined in our article. In other words, 73.41% of the target successfully hybridization with H1 and H2. The interaction matrix also lists other existing strands for reference, as indicated in the table.

Table S1. Interaction matrix for the proposed N1 targeted HCR.

Target	H1	H2	Conc. (µM)
1	1	1	0.7341
0	0	1	0.2658
1	1	0	0.1388
1	0	0	0.1267
0	1	0	0.1263

S2. Validation of the HCR Designer Using Gel Electrophoresis Data

As stated in the article, we use LN1 intensity profile of each HCR reaction to compare the hybridization efficiencies. In order to quantitatively compare the intensity of the gel lanes, ImageJ an image processer from NIH (https://image j.nih.gov/ij/) was used to analyze Figure 2.

To analyze the hybridization level of each HCR reactions in LN1, the cross-sectional profile of each lane was extracted. Result of the extraction is shown in Figure S3. Figure S3a reveals the procedures of the extraction. Three intensity profiles were extracted to calculate the final cross-sectional profile of a given HCR reaction. As shown in the figure, electrophoresis data profile and a baseline profile were firstly extracted. The baseline profile was then subtracted from the data profile, in order to take out the baseline intensity. The ladder intensity profile was also extracted from the image. Using the ladder profile, fluorescence data profile as a function of image pixel was then calibrated back into molecular weight (in unit of bp) axis. In this way, the molecular weights vs. fluorescence intensity profile for each HCR were finally obtained. We can therefore compare the intensity profile of each HCR reactions.

As shown in the Figure S3b, *N2* HCR exhibited almost no intensity above 100 bp molecular weight. *N1* HCR exhibit a noticeable level of product when the molecular weight is lower than 500 bp. With intensity profile stronger than *N1*, *N3* HCR exhibits some hybridization products with molecular weight larger than 500 bp. Finally, we can see clearly that the *RNase P* HCR products extended far beyond 500 bp. As a result, we have observed that the ratio of hybridization follows the trend of *RNase P* > *N3* > *N1* > *N2*.

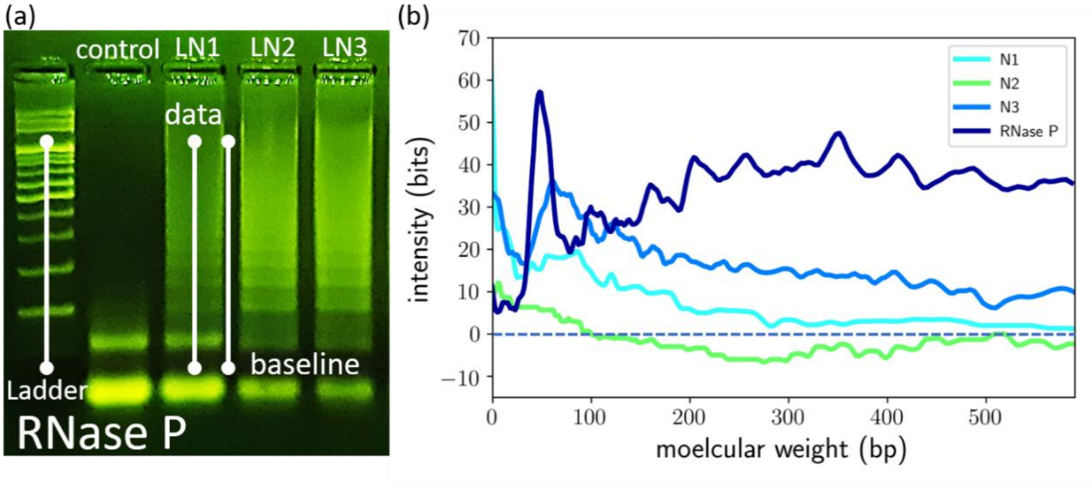

Figure S3. (**a**) extraction of intensity profile, using *RNase P* data as an example. (**b**) the LN1 fluorescence intensity profiles extracted from *N1*/*N2*/*N3*/*RNase P* gel electrophoresis results.
